# The novel dual GC-A and GC-B designer natriuretic peptide, cenderitide (CD-NP), enhances the renal actions of furosemide in a model of severe heart failure

**DOI:** 10.1186/1471-2210-11-S1-P17

**Published:** 2011-08-01

**Authors:** Lisa C Costello-Boerrigter, Guido Boerrigter, Sarah Mangiafico, Alessandro Cataliotti, John C Burnett

**Affiliations:** 1 Cardiorenal Research Laboratory, Mayo Clinic, Rochester, MN, USA

## Background

Patients with congestive heart failure (HF) have symptomatic improvement with diuretic therapy, although with time diuretic resistance and renal dysfunction can occur. Cenderitide (also known as CD-NP), now in clinical trials in patients with HF, is a Mayo designed chimeric natriuretic peptide which, unlike the native natriuretic peptides (NPs) ANP, BNP and CNP, binds both to guanylyl cyclase (GC)-B and GC-A with greater affinity for GC-B. Cenderitide thus was designed to mediate more venodilation than arterial dilation via GC-B and so result in less hypotension than BNP or ANP, but unlike CNP also possess natriuretic and diuretic properties via GC-A activation. We tested the hypothesis that combining cenderitide with furosemide will produce increased diuresis and natriuresis compared to furosemide alone without causing excessive hypotension or renal dysfunction in experimental HF.

## Methods

HF was induced in two groups of dogs (n=3 each) by tachypacing. On day 11 of pacing an acute study was performed under general anesthesia. The left ureter was cannulated and the renal artery was equipped with a flow probe. A continuous inulin infusion was started to measure glomerular filtration rate (GFR) and after equilibration a 30-minute baseline clearance (C1) was done. After that, one group (F+cenderitide) received a 105-minute cenderitide infusion (100 ng/kg/min), while furosemide (1 mg/kg) was administered over 60 minutes. After a 15-minute lead-in, three 30-minute clearances were done (C2, C3, C4). For the other group (F) furosemide was administered together with vehicle instead of cenderitide. Changes from baseline were compared between groups by unpaired t-test. Values are mean±SEM. *p<0.05 between groups.

## Results

F+cenderitide compared to F resulted in greater increases in sodium excretion* (+993±123 vs +426±98 µEq/min) and urine flow* (+8.5±0.8* vs +4.1±0.5 mL/min) in C2 and cumulatively from C2 through C4. GFR, renal blood flow and renal vascular resistance were similar between groups. Addition of cenderitide to F reduced right atrial pressure* but did not result in systemic hypotension.

## Conclusion

Adding cenderitide to furosemide significantly increases natriuresis and diuresis as compared to furosemide alone. Also, compared to F alone F+cenderitide results in a greater reduction in cardiac preload without inducing systemic hypotension. These findings suggest that the combination of F+ cenderitide represents an innovative co-therapy to enhance renal response to diuretics and to augment preload reduction; therefore, warranting further studies in human HF.

